# Differential expression of ABCB5 in BRAF inhibitor-resistant melanoma cell lines

**DOI:** 10.1186/s12885-018-4583-3

**Published:** 2018-06-22

**Authors:** Jingjing Xiao, Michael E. Egger, Kelly M. McMasters, Hongying Hao

**Affiliations:** The Hiram C. Polk, Jr MD Department of Surgery, University of Louisville School of Medicine, Louisville, KY 40292 USA

**Keywords:** BRAF melanoma, ATP-binding cassette transporter 5 (ABCB5), Vemurafenib (PLX4032), Chemoresistance

## Abstract

**Background:**

More than 50% of metastatic melanoma patients have a specific mutation in the serine/threonine kinase BRAF. This results in constitutive activation of the RAS-RAF-MEK-ERK-MAP kinase pathway, which causes uncontrolled cell growth. Vemurafenib (PLX4032) is an oral chemotherapeutic agent that targets the specific mutation V600E in the BRAF protein. Initial response rates are high in patients with BRAF mutant melanoma treated with a BRAF inhibitor such as vemurafenib, but resistance nearly always develops and disease progression ensues. There are several different mechanisms by which melanoma develops BRAF inhibitor resistance. One potential component of resistance is increased drug efflux. Overexpressed ABCB5 (ATP-binding cassette transporter, subfamily B, member 5) has been shown to efflux anti-cancer drugs from cancer cells. The purpose of this study is to determine whether ABCB5 is highly expressed in BRAF inhibitor-resistant melanoma cells and to evaluate whether ABCB5 is involved in the development of resistance to BRAF inhibitors in cutaneous melanoma.

**Methods:**

We established three BRAF inhibitor-resistant melanoma cell lines with BRAF mutation. The expression level of ABCB5 in PLX-resistant cell lines was checked by real-time PCR and Western blot analysis. SK-MEL-2 melanoma cells with wild-type BRAF were used for comparison. The association of different levels of ABCB5 with the changes of ERK, p-ERK, Akt and p-Akt was also assessed by Western blotting. Re-sensitization of melanoma cells to PLX was tested by p-ERK inhibitor PD58059 and ABCB5 knockdown by ABCB5 siRNA, respectively.

**Results:**

We showed that ABCB5 was overexpressed in SK-MEL-28PLXr and A2058PLXr cells but not in A375PLXr cells. ABCB5 overexpression is associated with activation of p-ERK status but not Akt. Inhibition of p-ERK re-sensitized SK-MEL-28PLXr and A2058PLXr cells to PLX treatment, but knockdown of ABCB5 did not re-sensitize A2058 PLXr and SK-MEL-28 PLXr cells to PLX treatment.

**Conclusion:**

These results confirm that, even though ABCB5 was overexpressed in SK-MEL-28 and A2058 melanoma cells that develop resistance to BRAF inhibitors, ABCB5 may not be a major targetable contributor to BRAF resistance. p-ERK inhibition may play important roles in BRAF resistance in these two melanoma cell lines.

## Background

Cutaneous melanoma can be classified into four different genomic subtypes: mutant *BRAF*, mutant *RAS*, mutant *NF1*, and triple-WT (wild-type) [1]. The largest subtype is defined by the presence of BRAF hot-spot mutations [1]. Targeted therapies using BRAF inhibitors can successfully block oncogenic signaling in BRAF-mutant melanomas, leading to significant improvement in progression-free survival (PFS) and overall survival [2–4]. However, melanoma cells quickly develop resistance to BRAF inhibitors, leading to high relapse rates. Therapeutic resistance to BRAF inhibitors is a major obstacle to the therapy of patients with BRAF mutation [5]. The precise mechanisms that underlie the therapeutic resistance of melanomas are not well understood [5]. Deciphering the mechanisms underlying the resistance of melanomas to targeted chemotherapy could significantly improve the outcomes of current therapies and could suggest novel approaches to new therapies.

ATP-binding cassette (ABC) superfamily of active transporters is found in both prokaryotes and eukaryotes with multiple functions. ABCB5 (subfamily B, member 5) is a human ABC transporter encoded on chromosome 7p15.3 [6, 7]. ABCB5 is expressed in malignant melanoma initiating cells (MMIC), a subset of melanoma cells with a stem cell phenotype [8]. These cells are known to represent heterogeneous circulating melanoma cells at all disease stages and are responsible for melanoma tumor growth and progression [8]. Another function of these transporters in eukaryotes is to efflux anticancer drugs from cells. ABCB5 has been shown to be a chemoresistance gene in melanoma and colorectal cancer patients [9]. ABCB5 was reported to be associated with resistance to several chemotherapeutic agents for melanoma, such as doxorubicin, dacarbazine, and temozolomide [6, 10, 11]. Our previous studies have shown that ABCB5 was associated with a poor clinical outcome in melanoma patients with positive sentinel lymph nodes [12]. However, little is known about how ABCB5 contributes to BRAF inhibitor resistance. The purpose of this study was to clarify whether ABCB5 was involved in PLX resistance in melanoma cells. Our data indicate that ABCB5 was overexpressed in BRAF resistant SK-MEL-28PLXr and A2058PLXr melanoma cells but not BRAF-resistant A375PLXr cells. ABCB5 overexpression in BRAF-resistant cells was associated with activation of p-ERK status but not Akt and p-Akt status. Inhibition of p-ERK re-sensitized SK-MEL-28PLXr and A2058PLXr cells to PLX treatment, but knockdown of ABCB5 did not re-sensitize A2058 PLXr and SK-MEL-28 PLXr cells to PLX treatment. These results suggest that, even though ABCB5 was overexpressed in SK-MEL-28PLXr and A2058PLXr melanoma cells, ABCB5 may not be a critical mediator of BRAF inhibitor-resistant melanoma. Inhibition of p- ERK may play important roles in BRAF resistance in these two melanoma cell lines.

## Methods

### Cell lines and culture reagents

Three melanoma cell lines with BRAF mutation, A375 (ATCC® CRL-1619™), A2058 (ATCC® CRL-11147™), and SK-MEL-28 (ATCC® HTB-72 ™), as well as one melanoma cell line harboring wild-type BRAF, SK-MEL-2 (ATCC® HTB-68 ™), were obtained from the American Type Culture Collection (Rockville, MD). A375 and A2058 cells were maintained in Dulbecco’s Modified Eagle Medium (DMEM), and SK-MEL-2 and SK-MEL-28 cells were maintained in α minimal essential medium (α-MEM), supplemented with 10% fetal bovine serum (FBS) and penicillin (100 U/mL)/streptomycin (100 μg/mL).

### Establishment of BRAF inhibitor-resistant melanoma cell lines

**Fig. 1:**
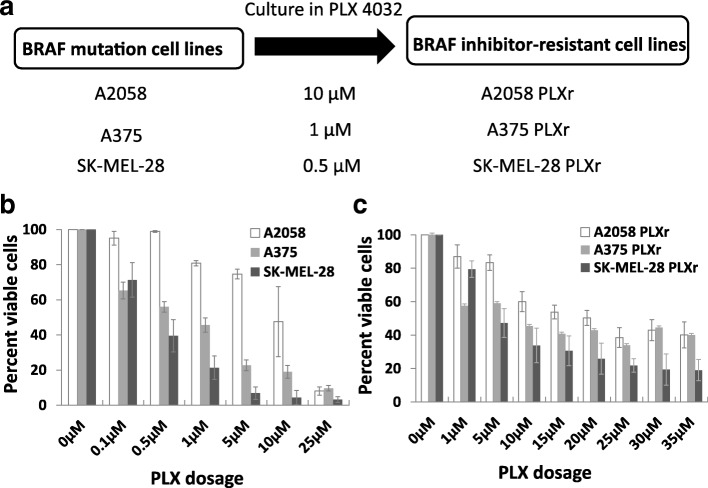
Discrepancy of sensitivity to PLX in different melanoma cell lines. **a** Schematic illustration of establishing PLX-resistant (PLXr) melanoma cell lines. Cell viability assay by MTT in melanoma cells (**b**) and PLX-resistant melanoma cells (**c**). Melanoma cells (**b**) and PLX-resistant melanoma cells (**c**) were treated for 72 h with different concentrations of PLX as indicated. Results are expressed as the percent of viable cells, and values represent the means ± SD (bars) of three independent experiments

BRAF inhibitor-resistant cell lines were established by adding 0.5 μM, 1.0 μM, and 10 μM of vemurafenib (PLX4032) in the culture media in SK-MEL-28, A375, and A2058 cells, respectively. The cells were passaged twice every week for 1 year to induce BRAF inhibitor resistance. Figure 1a shows the schematics of how the PLX-resistant cell lines were created. Maintenance of vemurafenib resistance was evaluated monthly via cell viability assay. The PLX-resistant cells were denoted as SK-MEL-28PLXr, A375PLXr, and A2058PLXr. Vemurafenib (PLX4032) was purchased from Selleck Chemicals (Houston, TX). PD98059 and temozolomide (TMZ) were purchased from Sigma-Aldrich (St. Louis, MO). The stock solutions of PLX at 10 mM, TMZ at 100 mM, and PD98059 at 50 mM were prepared in dimethyl sulfoxide and stored at -20 °C.

### Cell growth inhibition by 3-(4,5-dimethylthiazol-2-yl)-2,5-diphenyltetrazolium bromide assay

Cell lines were seeded in 48-well culture plates. After 24 h of incubation, cells were treated with PLX or TMZ as indicated. After treatment, melanoma cells were incubated with 3-(4,5-dimethylthiazol-2-yl)-2,5-diphenyltetrazolium bromide (MTT; 0.5 mg/mL, Sigma Aldrich) at 37 °C for 4 h according to the manufacturer’s instructions. After incubation, 500 μL of lysis buffer (10% SDS in 0.01 N HCl) was added, and samples were incubated at 37 °C overnight for complete cell lysis. On the following day, cytotoxicity was assessed by measuring the conversion of MTT to formazan through measurement of absorbance at a wavelength of 570 nm. The results are expressed as the percentage of viable cells from triplicate samples.

### RNA isolation and mRNA expression by semi-quantitative reverse transcription–PCR

mirVana RNA isolation kit (Life Technologies) was used to isolate total RNAs from cells following the manufacturer’s guidelines. Nanodrop ND-1000 (Thermo Fisher Scientific) was used to quantify total RNA. One thousand nanograms of total RNA from each cell line was reverse transcribed with the SuperScript III First-Strand Synthesis System for RT-PCR (Life Technologies). mRNA primers were purchased from Life Technologies. 7500 Fast Real-Time PCR System (Life Technologies) was used for RT-PCR reactions. Each target mRNA was normalized to an endogenous control gene (B_2_M). Fold changes were calculated with the 2^-ΔΔCt^ method.

### Western blotting

Cells were treated as indicated. Cell protein lysates were extracted in RIPA buffer and quantified by a BCA protein assay kit per manufacturer’s protocol (Thermo Fischer Scientific). Equal amounts of protein were loaded to electrophoresis and transferred to a nitrocellulose membrane. Membranes were probed at 4 °C overnight with primary antibody followed by probing with the appropriate secondary antibody conjugated to the horseradish peroxidase (Cell Signaling Technology, Danvers, MA). The primary human antibodies used were ABCB5 (abcam, ab140667, 1:1000 dilution), ERK (Cat #4695, Cell Signaling Technology, 1:1000 dilution), Akt (Cat #4685, Cell Signaling Technology, 1:1000 dilution), p-ERK (Cat #9106, Cell Signaling Technology, 1:500 dilution), and p-Akt (AF887, R&D Systems, 1:300 dilution). Equal loading of proteins was verified by probing the membrane again with an actin primary antibody (abcam, ab6276, 1:8000 dilution). Protein bands on film from Western blots were scanned, and the optical density of the bands were quantified using the NIH Image J software.

### Transfection of siRNA

A2058 PLXr or SK-MEL-28 PLXr cells were seeded in 24-well (1 × 10^5^ of cells per well) or 48-well (5 × 10^4^ of cells per well) and transfected with control siRNA or ABCB5 siRNA (Life Technologies) using DharmaFECT1 transfection reagent (Fischer Scientific) according to manufacturer’s protocol. Cells were subjected to PLX treatment as designated. Cell viability assay were performed as described above.

### Statistical analyses

Data are presented as representative images or as mean ± SD. The difference between groups was analyzed by a two-tailed Student *t*-test as applicable. A *p*-value of < 0.05 was considered to be statistically significant.

## Results

### Discrepancy of sensitivity to PLX in different melanoma cell lines

Vemurafenib (PLX4032) targets BRAF V600E-mutant melanoma. All three melanoma cell lines, A2058, A375, and SK-MEL-28, harbor BRAF mutation. To check the responses of melanoma cells to PLX treatment, cells were treated with PLX for 72 h. Cell viability was determined by MTT assay. The effect of PLX on the growth of melanoma cells at different concentrations is shown in Fig. 1b. SK-MEL-28 was most sensitive to PLX treatment, whereas A2058 was most resistant to PLX treatment. The IC50 of PLX of A2058, A375, and SK-MEL-28 cells was 10 μM, 1.0 μM, and 0.5 μM. The three cell lines, A2058, A375, and SK-MEL-28, were then constantly grown with the additional treatment of 10 μM, 1.0 μM, and 0.5 μM PLX. The cells were passaged twice every week for 1 year to induce BRAF inhibitor resistance and establish PLX-resistant (PLXr) cell lines. The vemurafenib-resistant cell lines were denoted as A2058PLXr, A375PLXr, and SK-MEL-28PLXr, respectively. The cell viability of PLX-resistant cell lines was determined by MTT assay (Fig. 1c). The IC50 of PLX of A2058 PLXr, A375 PLXr, and SK-MEL-28 PLXr were 15 μM, 10 μM, and 5 μM, respectively. The results showed that all three cell lines had developed resistance to BRAF inhibition with different levels of sensitivity.

### ABCB5 was differentially expressed in BRAF inhibitor-resistant cell lines

**Fig. 2:**
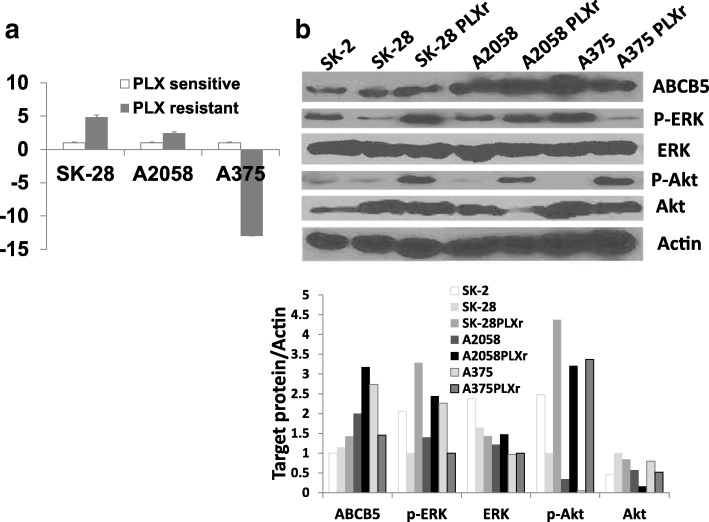
Differential expression of ABCB5, ERK, Akt, p-ERK, and p-AKT in wild-type BRAF cell, PLX-resistant melanoma cells and their parent cells by real-time PCR (**a**) and Western blot analysis (**b**). **a** Total RNA from melanoma cells and PLX-resistant melanoma cells were isolated. Real-time PCR was performed as described in Materials and Methods. B2M was used as an internal control. The bar graphs are expressed as fold changes of PLX resistant cell lines relative to each of their parent cell lines adjusted for B2M. Each of the experiments is a representation of two independent experiments performed in triplicate (** *p* < 0.01, *n* = 3). **b** Western blot analysis of ABCB5, ERK, Akt, p-ERK, p-Akt expression in wild-type BRAF cells, PLX-resistant cell lines, and their parent cell lines are shown. Anti-actin was used to show the equal loading and transfer in each lane. Targeted protein/actin showed the ratio of the scanned optical density of each protein band. ABCB5 protein level increased in A2058 PLXr and SK-MEL-28 PLXr cells versus A2058 and SK-MEL-28 cells. However, ABCB5 protein level was decreased in A375 PLXr cells versus A375 cells

ABCB5 is one of the most common genes that contribute to chemoresistance. To clarify whether ABCB5 was involved in PLX resistance in melanoma cells, the expression of ABCB5 mRNA was compared in PLX-resistant melanoma cell lines versus their parent melanoma cell lines by real-time PCR. The results showed that ABCB5 mRNA was upregulated in A2058 PLXr and SK-MEL-28 PLXr cells compared to A2058 and SK-MEL-28 cells (Fig. 2a); however, ABCB5 mRNA was downregulated in A375 PLXr cells compared to the A375 cells (Fig. 2a). We then examined the protein expression of ABCB5 by Western blotting. We also compared the expression of ABCB5 in the three parent cells that have BRAF mutation with that of another melanoma cell line, SK-MEL-2, which has wild-type BRAF expression. The results showed that SK-MEL-2 cells have a lower ABCB5 expression than the other three melanoma cell lines with BRAF mutation. ABCB5 protein level was upregulated in A2058 PLXr and SK-MEL-28 PLXr cells versus A2058 and SK-MEL-28 cells (Fig. 2b); however, ABCB5 protein level was downregulated in A375 PLXr cells versus A375 cells (Fig. 2b). The differential expression of ABCB5 protein was consistent with the differential expression of ABCB5 mRNA in the three PLX-resistant melanoma cell lines. These results suggest that not all the PLX-resistant melanoma cells have elevated ABCB5 expression. ABCB5 may be associated with resistance in some but not all BRAF inhibitor-resistant melanoma cell lines.

### Differential expression of ABCB5 in TMZ-treated and BRAF inhibitor-treated melanoma cells

**Fig. 3:**
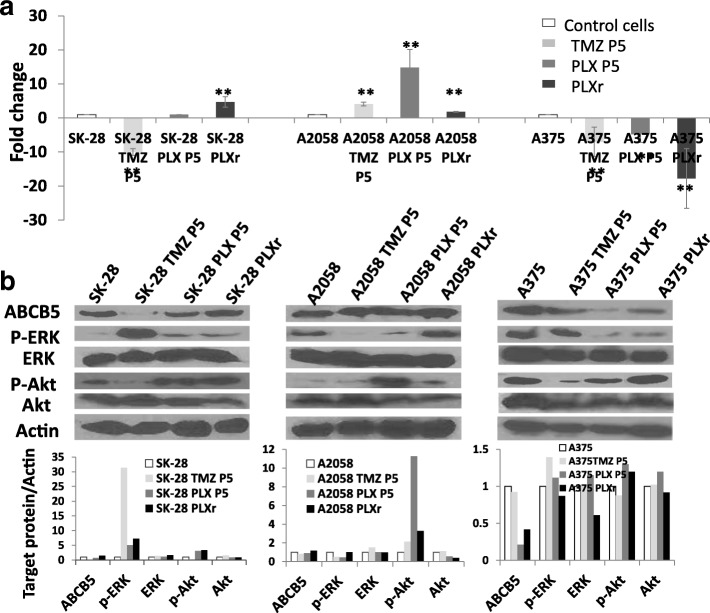
Differential expression of ABCB5, ERK, Akt, p-ERK, and p-Akt in TMZ-treated and PLX-treated melanoma cells. **a** Total RNA from TMZ-treated and PLX-treated melanoma cells were isolated. Real-time PCR was performed as described in Materials and Methods. B2M was used as an internal control. The bar graphs show expressed fold changes of PLX-resistant cell lines relative to each of their parent cell lines adjusted for B2M. Each of the experiments is a representation of two independent experiments performed in triplicate (** *p* < 0.01, *n* = 3). TMZ P5: Passage 5 of TMZ-treated melanoma cells. PLX P5: Passage 5 of PLX-treated melanoma cells. **b** Differential expression of ABCB5, ERK, Akt, p-ERK, and p-Akt in early passages of TMZ-treated and PLX-treated cells by Western blot analysis. Targeted protein/actin showed the ratio of the scanned optical density of each protein band. TMZ P5: Passage 5 of TMZ-treated melanoma cells. PLX P5: Passage 5 of PLX-treated melanoma cells

Unlike other chemotherapeutic reagents, PLX specifically targets BRAF mutation. We questioned whether there were different mechanisms between BRAF inhibitor-resistant cells and other chemoresistant cells. Temozolomide (TMZ) is another commonly used chemotherapeutic reagent in melanoma that has different mechanisms with PLX. According to our previous publication [13], human melanoma cells have variable sensitivity to TMZ in vitro. The IC50 of TMZ in SK-MEL-28, A2058, and A375 was 0.8 mM, 0.4 mM, and 0.4 mM. The dosages at IC50 of TMZ and PLX were used to treat in SK-MEL-28, A2058 and A375 cells. We compared the expression of ABCB5 in early passages of PLX-treated cells (passage 5, PLX P5) with that in early passages of TMZ-treated cells (passage 5, TMZ P5). The PCR results showed that ABCB5 mRNA had 4-fold increases in A2058 cells after TMZ treatment, whereas ABCB5 mRNA in SK-MEL-28 and A375 cells had 10-fold and 7-fold decreases after TMZ treatment (Fig. 3a). In passage 5 of PLX-treated cells, PCR results showed that ABCB5 mRNA increased significantly (14.8-fold) in A2058 cells. ABCB5 had marginal changes in SK-MEL-28 PLX P5 cells compared with SK-MEL-28 cells. ABCB5 had 4.7-fold decreases in A375 PLX P5 cells when compared with A375 cells (Fig. 3a). We also checked the ABCB5 protein expression in early passages of PLX-treated cells (passage 5, PLX P5) with that in early passages of TMZ-treated cells (passage 5, TMZ P5) by Western blotting. The results showed that the changes of the expression level of ABCB5 protein were mostly consistent with those of the ABCB5 mRNA, although the ratios were not exactly matched with the fold changes of the PCR (Fig. 3b). These results demonstrate that ABCB5 was differentially expressed in TMZ-treated and PLX-treated melanoma cells. ABCB5 might have different roles in response to different chemotherapeutic agents.

Previous research reported that chemotherapy leads to the selection of ABCB5-expression cells [11]. We sought to identify what changes occur in ABCB5 within early passages of PLX-resistant cells as compared with the established PLX-resistant cell lines. We collected early passages of PLX cell lines and compared the ABCB5 expression in early passages of PLX-resistant cells with the established PLX-resistant cells by real-time PCR. As shown in Fig. 3a, ABCB5 did not have significant changes in early passages of SK-MEL-28 PLX P5 cells, but ABCB5 had a considerable 4.7-fold increase in established SK-MEL-28 PLXr cells. In A2058 cells, ABCB5 had a drastic 14.8-fold increase in early passages of A2058 PLX P5 cells. In contrast, ABCB5 mRNA had a 1.8-fold increase in established A2058 PLXr cells. In A375 cells, ABCB5 was decreased at the early time point of PLX treatment, showing 2.4-fold downregulation in A375 PLX P5 cells versus A375 cells. The level of ABCB5 remained low (− 8 fold) in A375 PLXr cells. The changes of ABCB5 protein expression were similar as we observed in the PCR. These results showed that ABCB5 expression fluctuated in early passages of PLX-resistant cells and the established PLX-resistant cells. Based on these results, one may conclude that ABCB5 could respond differently during the course of chemotherapeutic treatment.

### Upregulation of ABCB5 in BRAF inhibitor-resistant melanoma cell lines was associated with upregulation of p-ERK

Reactivation of the extracellular signal-regulated kinase (RAF-ERK) pathway and activation of the PI3K pathway were two major mechanisms identified in BRAF resistance [14–17]. We questioned whether the differential expression of ABCB5 in BRAF inhibitor-resistant cell lines had any connection with the expression of ERK, p-ERK, Akt, and p-Akt status in those cells. Western blotting showed that ERK expression did not have significant changes in all three types of BRAF inhibitor-resistant cells versus non-resistant cells. In A2058 PLXr and SK-MEL-28 PLXr cells in which ABCB5 was overexpressed, p-ERK expression was also increased. On the other hand, in A375 PLXr cells in which ABCB5 expression decreased, p-ERK level was also reduced (Fig. 3b). Akt was downregulated in all three types of BRAF inhibitor-resistant cells versus non-resistant cells. Nevertheless, p-Akt was upregulated in all three types of BRAF inhibitor-resistant cells versus non-resistant cells (Fig. 3b). In SK-MEL-2 cells with wild-type BRAF, the ERK expression had no significant difference with the other three BRAF mutation cells. However, the Akt level was much lower in SK-MEL-2 cells compared with the other three BRAF mutation cells (Fig. 2b). These results indicated that upregulation of ABCB5 in BRAF inhibitor-resistant cell lines might be associated with the activation of p-ERK status.

### Akt was upregulated in early passages of TMZ-treated and BRAF inhibitor-treated cells

As we noted above, ABCB5 was differentially expressed in early passages of TMZ-treated and BRAF inhibitor-treated melanoma cells. We questioned whether there were any differences in the expression level of ERK and Akt in melanoma cells in response to different chemotherapeutic agents. Western blotting showed that ERK level nearly had no changes in early passages of either TMZ-treated or PLX-treated melanoma cells (Fig. 3b). However, unlike the downregulation of Akt in A2058 PLXr, A375 PLXr, and SK-MEL-28 PLXr cells versus their parent cells, Akt was increased in early passages of both TMZ-treated and PLX-treated melanoma cells. In contrast, Akt levels were decreased in all three BRAF inhibitor-resistant melanoma cells (Fig. 3b). These results suggested that Akt expression first increased as an early response to PLX treatment and then decreased as a late response to PLX treatment.

### Knockdown of ABCB5 did not re-sensitize A2058 PLXr, SK-MEL-28 PLXr or A375 cells to PLX treatment

**Fig. 4:**
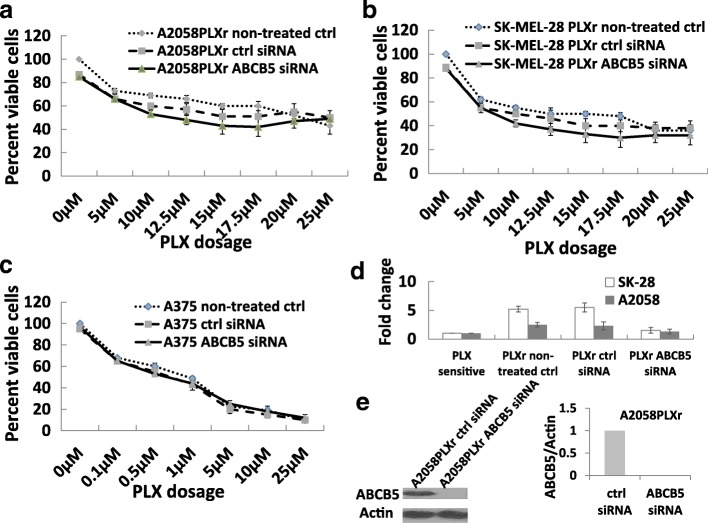
Knockdown of ABCB5 did not re-sensitize A2058 PLXr, SK-MEL-28 PLXr, and A375 cells to PLX treatment. A2058 PLXr cells (**a**), SK-MEL-28 PLXr cells (**b**), and A375 cells (**c**) were seeded in 24-well plates. Cells were transfected with either ABCB5 control siRNA or ABCB5 siRNA the next day. After 6 h of transfection, PLX was added to each well as designated. Cells that were not transfected were used as another group of control. Cell viability assay by MTT was performed after 72 h of PLX treatment. Results are expressed as the percent viable cells and values represent the means ± SD (bars) of three independent experiments. **d** A375 cells, PLX-resistant A2058 PLXr and SK-MEL-28 PLXr cells were seeded in 12-well plates. Cells were transfected with either ABCB5 control siRNA or ABCB5 siRNA the next day. After 12 h of transfection, total RNA was isolated. Real-time PCR was performed as described in Fig.2a (ctrl: control). **e** ABCB5 knockdown by ABCB5 siRNA was shown by Western blot in A2058PLXr cells. ABCB5/actin showed the ratio of the scanned optical density of the ABCB5 protein band

A375 parent cells had the highest level of ABCB5 expression among all the cells tested in this study. The expression of ABCB5 increased in A2058 PLXr and SK-MEL-28 PLXr cells versus A2058 and SK-MEL-28 cells. We questioned whether knockdown of ABCB5 might re-sensitize A2058 PLXr, SK-MEL-28 PLXr, and A375 cells to PLX treatment. A375, A2058 PLXr, and SK-MEL-28 PLXr cells were transfected with ABCB5 siRNA and then subjected to analysis of cell viability and a cytotoxicity assay. There were no significant changes of cell viability after PLX treatment in A2058 PLXr (Fig. 4a), SK-MEL-28 PLXr (Fig. 4b), and A375 cells (Fig. 4c) after control siRNA and ABCB5 siRNA transfection (Fig. 4d and e). These results showed that, even though ABCB5 was highly expressed in A375, A2058PLXr, and SK-MEL-28PLXr BRAF inhibitor-resistant cells, knockdown of ABCB5 did not re-sensitize these cells to PLX treatment. ABCB5 upregulation may not be the key player to BRAF inhibitor-resistance in A375, A2058PLXr, and SK-MEL-28PLXr cells.

### Inhibition of p-ERK re-sensitized SK-MEL-28PLXr and A2058PLXr cells to PLX treatment

**Fig. 5:**
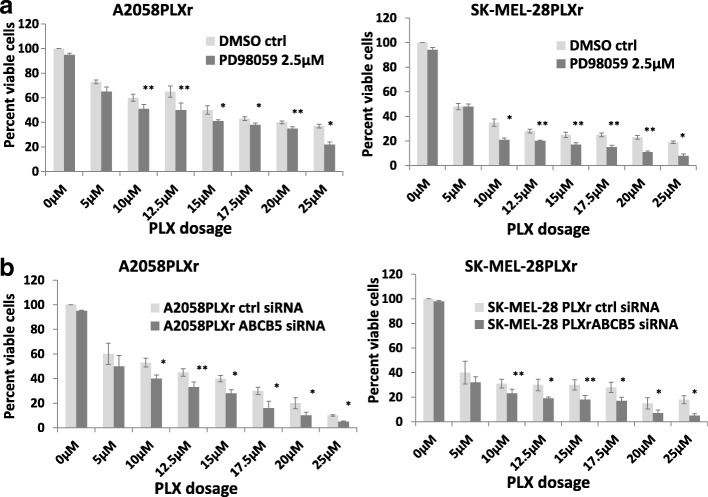
Re-sensitization of SK-MEL-28PLXr and A2058PLXr cells to PLX treatment by p-ERK inhibitor PD98059. **a** SK-MEL-28PLXr cells and A2058PLXr cells were treated by PD98059 and PLX for 72 h as indicated. Cell viability assay was performed by MTT. Results are expressed as the percent of viable cells, and values represent the means ± SD (bars) of three independent experiments. **b** A2058 PLXr cells and SK-MEL-28 PLXr cells were seeded in 48-well plates. Cells were treated by PD98059 as indicated. Cells were transfected with either ABCB5 control siRNA or ABCB5 siRNA the next day. After 6 h of transfection, PLX was added to each well as designated. Cell viability assay was performed by MTT after 72 h of transfection. Results are expressed as the percent of viable cells, and values represent the means ± SD (bars) of three independent experiments (*p < 0.05, ** *p* < 0.01, *n* = 3)

Since p-ERK were upregulated after SK-MEL-28 and A2058 had developed resistance to BRAF inhibitor PLX, we were interested in knowing whether inhibition of p-ERK can re-sensitize SK-MEL28PLXr and A2058 PLXr cells to PLX treatment. We used PD98059, a selective p-ERK inhibitor, to treat these two PLX-resistant cell lines. Our initial data showed that PD98059 does not cause significant cell death at the dose of 2.5uM (data not shown). We used this dose to test the cell viability after PLX treatment. The results showed that inhibition of p-ERK can re-sensitize SK-MEL-28PLXr and A2058PLXr cells to PLX treatment (Fig. 5a). We then checked whether knockdown of ABCB5 in combination with inhibition of p-ERK could have a synergistic effect on re-sensitizing SK-MEL-28PLXr and A2058PLXr cells to PLX treatment. The results showed that knockdown of ABCB5 in combination with inhibition of p-ERK can also re-sensitize PLXr cells to PLX treatment, but they did not have a synergistic effect as compared to inhibition of p-ERK alone (Fig.5b). These results suggested that inhibition of p- ERK may play a more important role than the repression of ABCB5 in PLX resistance in these two melanoma cell lines.

## Discussion

ABCB5, a member of ABC transporter family, was cloned by Frank and colleagues in 2003 [7]. Subsequently, ABCB5 was identified as a potential predictor of active recurrence/progression events, with its expression in blood correlating with disease recurrence and progression in melanoma patients [18]. Our previous research has also shown that ABCB5 was highly expressed in the sentinel lymph nodes of melanoma patients who experienced recurrence [12]. These findings prompted us to hypothesize that ABCB5 might contribute to melanoma recurrence through resistance to chemotherapeutic drugs. There are some reports about the expression of ABCB5 in connection with the chemoresistance. Side population (SP) cells are chemoresistant cells present in human melanoma. These cells overexpress ABCB5 and are capable of excluding anticancer drugs [19]. Chartrain et al. showed that ABCB5-expressing cells selectively survive when exposed to dacarbazine, vemurafenib, and other various chemotherapeutic drugs in WM-266-4, G-361, and SK-MEL-28 cells [11]. This research group also reported that melanoma patients had enriched ABCB5-expressing cells in vivo after clinical TMZ therapy [11]. Our current study also showed that ABCB5 was overexpressed in two of the BRAF inhibitor-resistant melanoma cells (A2058 PLXr and SK-MEL-28 PLXr) but not A375 PLXr cells. At the initial treatment of PLX in A2058, SK-MEL-28, and A375 cells, ABCB5 increased significantly in the early passages of A2058 cells, decreased considerably in the early passages of A375 cells, and remained stable in early passages of SK-MEL-28 cells. In response to TMZ treatment in these three melanoma cell lines, ABCB5 increased significantly in A2058 cells but decreased extensively in SK-MEL-28 and A375 cells. These data suggested that ABCB5 was differentially expressed in response to different chemotherapeutic drugs in different melanoma cells. In addition, the changes in ABCB5 differed between the early passages and the late passages of melanoma cells in response to BRAF treatment. This highlights a very important factor in tailoring chemotherapeutic strategies in different individuals and during the different treatment stages of each individual patient in clinical practice.

In the three BRAF-resistant cell lines that were tested, all had a relatively higher expression of ABCB5 than the wild-type BRAF cell line SK-MEL-2. The Akt level in SK-MEL-2 cells was markedly lower than that of the other three BRAF mutation cells; however, since there is only one wild-type BRAF cell line in this study, generalization of this conclusion to all the BRAF wild-type cells and BRAF mutation cells is not feasible. Whether or not constitutive activation of ABCB5 is required for vemurafenib resistance needs to be further studied.

Extensive research has been conducted on deciphering the roles of ABCB5 in chemoresistance. ABCB5 is shown as a functional drug transporter and chemoresistance mediator to multiple chemotherapeutic agents such as doxorubicin [6, 20, 21]. Inhibition of ABCB5 can increase intracellular drug accumulation and confer melanoma cells’ sensitivity to doxorubicin [6]. However, little has been studied regarding the roles of ABCB5 on BRAF inhibitor-resistant melanoma cells. Our current study showed that, even though ABCB5 was overexpressed in A2058PLXr and SK-MEL-28PLXr cells, knockdown of ABCB5 by siRNA did not re-sensitize the cells to PLX treatment. This suggests that ABCB5 may not be a major contributor to BRAF resistance in A2058 PLXr and SK-MEL-28 PLXr cells. Our study provides different insights into the function of ABCB5 in melanoma chemoresistance to BRAF inhibitor. Similar to our findings, Vasquez-Moctezuma et al. [22] found that not all melanomas are positive for ABCB5 expression. They showed that ABCB5 expressing melanomas had variable gene expression and suggested that the ABCB5 gene may be differentially regulated by individual melanomas.

One possible explanation for these varying responses is that development of multidrug resistance depends on the expression and function of various genes in cells. ABCB5 may not be the single contributor to chemoresistance. Gerber et al. [8] used single-cell RNA-seq in patient-derived melanoma cultures and found that about 10% of all the *BRAF*/*NRAS* wild-type cells co-expressed ABC transporter family with aldehyde dehydrogenases (ALDHs). About 20–40% of cells in the mutant cells (*BRAF* wild-type/*NRAS* mutant and *BRAF* mutant/*NRAS* wild-type) have co-expression of ABC transporters along with ALDHs. Co-expression of ABCB5 with ALDHs may support their possible roles in resistance against chemotherapy [8]. Another research study from the Gottsman group showed that melanosomes contribute to the refractory properties of melanoma cells by sequestering cytotoxic drugs and increasing melanosome-mediated drug export [23]. They suggested that the dynamics of melanosome (including their structural integrity, density, and biogenesis) can adjust the drug resistance of melanoma cells [24]. All of these data support the fact that ABCB5 may not directly potentiate chemoresistance, but may be responsible for increasing heterogeneity in the cancer cell population [25]. Deliberately disrupting or inhibiting ABCB5 in melanomas may not be sufficient to improve the therapeutic resistance.

There are two major pathways that are involved in BRAF resistance. One is MAPK-dependent pathway and the other is MAPK-independent mechanism. MAPK-dependent pathway mainly involves reactivation of the MAPK pathway to substitute the suppression of BRAFV600E. This can be acquired through several mechanisms, such as amplification of BRAFV600E, expression of alternative splicing forms of BRAFV600E, or acquisition of activating mutations in NRAS or MEK (MAP2K1) [15, 26–28]. Another alternative path to BRAF resistance is the enhanced signaling through the PI3K/AKT pathway, with or without concomitant MAPK reactivation [29]. AKT signaling mechanism is mediated by several genetic changes. These include elevated expression of IGF1R (insulin-like growth factor 1 receptor) and HGF (hepatocyte growth factor) by stromal cells. They all have been linked to BRAF inhibitor resistance [17, 30, 31]. Other mediators of BRAF resistance have also been reported, such as upregulation of the PDGFRB (tyrosine kinase platelet-derived growth factor receptor beta), possibly through PI3K- or MAPK-related mechanisms [15]. Understanding the pathways involved in BRAF resistance and their relationship with ABCB5 expression may help define and develop potential drug targets. In doxorubicin-resistant breast cancer cells that have high levels of ABCB5, ERK-3 serine/threonine kinase is specifically upregulated, suggesting that ABCB5 and ERK3 could be potential targets against drug-resistant breast cancer cells [25]. In our study, we found that ERK expression was consistent in all three types of BRAF inhibitor-resistant cells versus non-resistant cells. In A2058 PLXr and SK-MEL-28 PLXr cells in which ABCB5 was overexpressed, p-ERK expression was also increased. Nonetheless, in A375 PLXr cells in which ABCB5 was downregulated, p-ERK levels decreased. Akt was downregulated and p-Akt was upregulated in all three types of BRAF inhibitor-resistant cells versus non-resistant cells. These results suggest that overexpression of ABCB5 in BRAF inhibitor-resistant melanoma cell lines was associated with upregulation of p-ERK.

Further studies with a p-ERK inhibitor, PD98059, confirmed that inhibition of p-ERK can reverse the BRAF inhibition in those BRAF inhibitor-resistant melanoma cells. However, knockdown of ABCB5 in combination with inhibition of p-ERK did not have a synergistic effect on re-sensitizing the PLX-resistant cells to PLX treatment as compared to inhibition of p-ERK alone. These results may imply that inhibition of p- ERK contributes more than the suppression of ABCB5 in reversion of PLX resistance in melanoma cells. This also suggests that constitutive activation of ABCB5 may not be required for vemurafenib resistance.

## Conclusions

ABCB5 was differentially expressed in response to different chemotherapeutic drugs in different melanoma cells. In addition, the changes of ABCB5 differed between the early passages and the late passages of melanoma cells in response to BRAF inhibitor treatment. Deliberately disrupting or inhibiting ABCB5 in melanomas might not be sufficient to improve the therapeutic resistance to BRAF inhibitors. Nevertheless, inhibition of p-ERK may re-sensitize the melanoma cells to the treatment of BRAF inhibitors. Our work provides further insight into the role of ABCB5 in BRAF inhibitor-resistant melanoma cells.

## Abbreviations

ABCB5: ATP-binding cassette transporter, subfamily B, member 5
ALDHs: Aldehyde dehydrogenases
ERK: Extracellular signal-regulated kinases
HGF: Hepatocyte growth factor
IGF1R: Insulin-like growth factor 1 receptor
MAP: Mitogen-activated protein
MMIC: Malignant melanoma initiating cells
MTT: 3-(4, 5-dimethylthiazol-2-yl)-2,5-diphenyltetrazolium bromide
PDGFRB: Platelet-derived growth factor receptor beta
PFS: Progression-free survival
PLX4032: Vemurafenib
RAF: Rapidly accelerated fibrosarcoma
RAS: Retrovirus-associated DNA sequences
SP: Side population
TMZ: Temozolomide
WT: Wild-type
